# Concurrent validity and reliability of the Community Balance and Mobility scale in young-older adults

**DOI:** 10.1186/s12877-018-0845-9

**Published:** 2018-07-03

**Authors:** Michaela Weber, Jeanine Van Ancum, Ronny Bergquist, Kristin Taraldsen, Katharina Gordt, A. Stefanie Mikolaizak, Corinna Nerz, Mirjam Pijnappels, Nini H. Jonkman, Andrea B. Maier, Jorunn L. Helbostad, Beatrix Vereijken, Clemens Becker, Michael Schwenk

**Affiliations:** 1 Network Aging Research (NAR), Heidelberg University, Bergheimer Straße 20, DE-69115 Heidelberg, Germany; 2 Department of Human Movement Sciences, Amsterdam Movement Sciences, Vrije Universiteit Amsterdam, Amsterdam, The Netherlands; 3 Department of Medicine and Aged Care, @AgeMelbourne, Royal Melbourne Hospital, University of Melbourne, Melbourne, Australia; 4 Department of Neuromedicine and Movement Science, Norwegian University of Science and Technology, Trondheim, Norway; 5 Department of Clinical Gerontology, Robert-Bosch Hospital, Stuttgart, Germany

**Keywords:** Aging, Balance, Mobility, Physical performance, Assessment, Measurement properties, Older adults

## Abstract

**Background:**

With the growing number of young-older adults (baby-boomers), there is an increasing demand for assessment tools specific for this population, which are able to detect subtle balance and mobility deficits. Various balance and mobility tests already exist, but suffer from ceiling effects in higher functioning older adults. A reliable and valid challenging balance and mobility test is critical to determine a young-older adult’s balance and mobility performance and to timely initiate preventive interventions. The aim was to evaluate the concurrent validity, inter- and intrarater reliability, internal consistency, and ceiling effects of a challenging balance and mobility scale, the Community Balance and Mobility Scale (CBM), in young-older adults aged 60 to 70 years.

**Methods:**

Fifty-one participants aged 66.4 ± 2.7 years (range, 60–70 years) were assessed with the CBM. The Fullerton Advanced Balance scale (FAB), 3-Meter Tandem Walk (3MTW), 8-level balance scale, Timed-Up-and-Go (TUG), and 7-m habitual gait speed were used to estimate concurrent validity, examined by Spearman correlation coefficient (*ρ*). Inter- and intrarater reliability were calculated as Intra-class-correlations (ICC), and internal consistency by Cronbach alpha and item-total correlations (*ρ*). Ceiling effects were determined by obtaining the percentage of participants reaching the highest possible score.

**Results:**

The CBM significantly correlated with the FAB (*ρ* = 0.75; *p* < .001), 3MTW errors (*ρ* = − 0.61; *p* < .001), 3MTW time (*ρ* = − 0.35; *p* = .05), the 8-level balance scale (*ρ* = 0.35; *p* < .05), the TUG (*ρ* = − 0.42; *p* < .01), and 7-m habitual gait speed (*ρ* = 0.46, *p* < .001). Inter- (ICC_2,k_ = 0.97), intrarater reliability (ICC_3,k_ = 1.00) were excellent, and internal consistency (*α* = 0.88; *ρ* = 0.28–0.81) was good to satisfactory. The CBM did not show ceiling effects in contrast to other scales.

**Conclusions:**

Concurrent validity of the CBM was good when compared to the FAB and moderate to good when compared to other measures of balance and mobility. Based on this study, the CBM can be recommended to measure balance and mobility performance in the specific population of young-older adults.

**Trial registration:**

Trial number: ISRCTN37750605. (Registered on 21/04/2016).

## Background

Balance ability generally starts to decline in the third decade of life [1], with an accelerated decline occurring in the sixth decade [2, 3]. Older adults (≥65 years) are more prone to experience a loss of function preventing them to maintain posture and respond to unexpected perturbations caused by slips or trips [4]. Young-older adults of retirement age (60–69 years [5]) generally function at a higher level compared to (old-) older adults. However, their more active lifestyle potentially exposes them to more high-risk balance-challenging situations. Subsequently, the risk for stumbles and near-falls is significantly higher [6]. With a dramatic increase in the proportion of young-older adults (baby boomer generation), a paradigm shift is requested towards early stage innovative population-level efforts to prevent loss of balance [7].

Regular physical activity (PA) is important to maintain independence and prevent functional decline. Current guidelines for older adults aged ≥65 years recommend at least 150 min of moderate intensity or 75 min of vigorous intensity aerobic training per week [8]. Persons with poor mobility should undertake training three or more days per week to improve balance and prevent falls [8]. However, less than 50% of older adults meet the current PA recommended [9] and only 6% complete regular balance training [10].

In order to promote early balance and mobility interventions, adequate assessment strategies are needed to identify subtle balance and mobility deficits in relatively active, high-functioning young-older adults. To date, most balance and mobility assessment tools have been developed to quantify deficits in frail older adults aged ≥70 years [11–16]. Current systematic reviews focusing on functional balance assessment have shown that several assessment tools developed for older adults are not appropriate for detecting early balance and/or gait deficits in community-dwelling older adults with a more active lifestyle [17, 18]. For example, the Berg Balance Scale (BBS), a widely-used, valid and reliable test of functional balance in frail older adults aged ≥70 years [12, 18]. This test reached ceiling effects when used in community-dwelling older adults aged ≥60 years [15, 17, 18]. With most of the items focusing on basic functional mobility (e.g. transfers, standing unsupported, sit-to-stand), the BBS does not include challenging dynamic balance tasks such as tandem walking, hopping, or climbing stairs. Likewise, the Short Physical Performance Battery (SPPB) was initially developed for community-dwelling older adults aged ≥70 years [19]. This test has also shown ceiling effects in higher-functioning community-dwelling older adults aged ≥60 years [15, 20]. Ceiling effects of these instruments do not only hamper the detection of early balance deficits, but also prevent the detection of intervention-related changes over time in higher functioning older adults [20, 21].

Current systematic reviews focusing on mobility in older adults conclude that tests such as the Timed Up and Go (TUG) test, the Dynamic Gait Index (DGI), or the Performance Oriented Mobility Assessment also suffer from ceiling effects when applied in independently living, higher functioning older adults [13, 17]. They are not challenging enough to adequately assess the performance of older adults who do not display marked mobility deficits, because they lack more demanding mobility components such as turning the head while walking [11, 13, 14, 17, 22].

In summary, several studies have shown that balance and mobility measures developed for older, frailer adults show ceiling effects when applied in high-functioning older adults [13, 15, 17, 18, 20, 23]. The lack of high-challenging balance tasks in the aforementioned scales can result in early signs of balance and mobility decline to remain unidentified. This makes the currently available balance and mobility tests less suitable when the aim is to determine intervention eligibility aimed at preventing decline in balance and mobility at an early stage [13, 24, 25].

In this context, the applicability of the Community Balance and Mobility Scale (CBM) has recently generated significant interest in clinical practice for assessing balance and mobility deficits in community-dwelling older adults, either healthy (mean age 70.3 years [26]) or with knee osteoarthritis (mean age 62.5 years [27]). Unlike commonly used balance and mobility tests such as the BBS [12], SPPB [19] or the Tinetti test [14], the CBM includes several challenging tasks to assess specific aspects of balance and mobility which are necessary to function independently within the community. For example, walking while gaze shifting and turning the head, picking up an object from the floor (crouching) while walking, and complex walking maneuvers, such as forward to backward walking, sideways walking, or suddenly stopping, are included in the CBM [28, 29]. The CBM was initially developed to measure subtle balance deficits in patients with mild traumatic brain injury aged 26.2 years [30] to 31.0 years and is found to be valid and reliable in this population [28, 30].

Recently, the CBM has been validated in a sample of independently living, community-dwelling older adults aged ≥65 years (mean age 73 ± 7), showing excellent correlations with the BBS (*ρ* = 0.87), good correlations with the Timed Up and Go test (*ρ* = − 0.69) and self-selected gait speed (*ρ* = − 0.65) [26]. Reliability of the rating scheme was also analyzed based on videotaped assessments resulting in high inter- (ICC_2,k_ = 0.95; 95% CI = 0.88–0.98) and intrarater reliability (ICC_3,k_ = 0.96; 95% CI = 0.93–0.98) [26]. Moreover, the CBM showed no ceiling effects as compared to BBS (23%) and SPPB (33%) [26].

While these findings suggest that the CBM has added value in the assessment of community-dwelling older adults, the measurement properties in the specific population of young-older adults aged 60–70 years are yet to be evaluated. Young-older adults are an extremely heterogeneous population, where some older adults have substantial balance and mobility deficits while others have only minor deterioration in balance performances [31]. The CBM may represent a specific assessment tool for detecting both minor and major balance and mobility deficits in this population, and in turn may allow early interventions to be tailored to prevent functional decline.

In this study, we aimed to examine the concurrent validity and reliability of the CBM in community-dwelling healthy young-older adults (60 to 70 years). The evaluation was performed as preparatory part of the European Commission funded project PreventIT (Horizon 2020 grant no 689238), which aims to develop a lifestyle-integrated training intervention to prevent functional decline in young-older adults.

The first aim of the present study was to examine the concurrent validity of the CBM by comparing its scores to other established balance and mobility measures thought to have related theoretical constructs. We expected a positive association with the Fullerton Advanced Balance Scale [32] as this scale has also been developed to measure balance problems of varying severity in functionally independent older adults. We expected a negative association with the Timed Up-and-Go test [33] based on previous validation studies in older adults [26, 27]. Furthermore, we hypothesized moderate to good associations with balance tests measuring static steady-state balance control (8-level balance scale, comprising the five level balance scale from the SPPB and additional challenging tasks at a higher level, such as “tandem stand eyes closed” [34]) and dynamic steady-state balance control (3 Meter Tandem Walking [34], and gait speed [26–28, 30, 35]). The second aim was to investigate the ceiling effects of the CBM as compared to other challenging balance and mobility assessments which, based on previous findings, were expected to be lower for the CBM [26, 27, 30]. The third aim was to investigate the intra- and interrater reliability of the rating scheme of the CBM, which was expected to be high based on previous studies in other populations [26, 28]. Finally, we aimed to analyze the internal consistency reliability.

## Methods

### Design

We used a cross-sectional study design for evaluating the concurrent validity and potential ceiling effects of the CBM. The inter- and intra-reliability was also obtained based on video-recordings of the assessments (described below). The data collection was embedded into the PreventIT project (phase 1). PreventIT is a three-year project aiming at developing a lifestyle-integrated training intervention for young-older adults aged 60 to 70 years. Phase 1 of the PreventIT project included pilot studies at the sites involved in the project (Stuttgart, Heidelberg, Amsterdam, and Trondheim). The pilot studies aimed to test the measurement properties of balance and mobility instruments in young-older adults. Another purpose of the PreventIT pilot studies was to test the feasibility of the lifestyle-integrated training intervention using questionnaires and focus groups. This feasibility testing occurred after the cross-sectional study for validating the CBM and did not influence this study.

### Participants

For the purpose of evaluating the measurement properties of the CBM in the specific population of young-older adults, we included 51 community-dwelling young-older adults. Inclusion criteria for this study were: community-dwelling older adults aged between 60 and 70 years, able to walk independently, and no cognitive impairment (Montreal Cognitive Assessment [36] ≥ 26 points). Participants were excluded if they reported severe cardiovascular, pulmonary, neurological, or mental disease. Participants were recruited for the pilot studies with the main purpose of examining a lifestyle-integrated training intervention in Germany (Robert-Bosch Hospital, Stuttgart; Heidelberg University), Norway (Norwegian University of Science and Technology), and the Netherlands (Vrije Universiteit Amsterdam). Ethical approval from the local institution review boards as well as written informed consent from participants were obtained in all four study centers prior to participation.

### Measures

Demographics and clinical variables were collected, including age, sex, body mass index, comorbidities, falls history in the previous year, and five performance-based assessment tests of balance and mobility as described in the following.

#### Balance and mobility assessments

The Fullerton Advanced Balance (FAB) scale is designed to identify balance deficits [32, 37] and has been validated in functionally independent older adults aged 75 ± 6 years with increased fall risk [32]. It includes 10 items scored from zero to four (higher values indicate better performance) with a maximum score of 40 points [32]. The tasks on the FAB are “Stand with feet together and eyes closed”, “Reach forward to retrieve a pencil held at shoulder height with outstretched arm”, “Turn 360 degrees in right and left directions”, “Step up onto and over a 6-inch bench”, “Tandem walk”, “Stand on one leg”, “Stand on foam with eyes closed”, “Two-footed jump”, “Walk with head turns”, and “Reactive postural control”.

The 8-level balance scale is an extended version of the SPPB [19] that incorporates several higher-level balance performance tasks [34]. The items are “Side-by-side Standing, narrow base Romberg” (eyes open; eyes closed), “Semi Tandem” (eyes open), “Tandem Stand” (eyes open; eyes closed), and “One Leg Stand” (eyes open; eyes closed; eyes closed with cognitive distractor). Participants have to complete successfully a balance task for 30 s before progressing to the next task. The highest level of balance test performed successfully was rated (maximum score: 8).

The three meter tandem walk (3MTW) test is a modified version of the FAB [32], measuring dynamic balance. The test requires participants to complete a three meter walk heel-toeing as quickly as possible, with as few errors as possible [34]. Number of errors during walking were defined as touching examiner or object in the environment, making a step with no heel-toe contact, or touching the ground in some other spot on the way to positioning the foot where it should be [34]. The time for completion (seconds) and the number of errors were recorded in a subsample (*n* = 31).

The Timed-Up-and-Go (TUG) test is a valid test evaluating basic functional mobility of older adults [33]. The test requires participants to stand up from a standard arm chair (45 cm height), walk three meters, turn around, walk back, and sit down again while being timed with a manual stopwatch [33, 38]. The time for completion (seconds) was recorded.

Gait speed measurement was derived from the InChianti gait assessment [35]. Participants are instructed to walk seven meters at their usual pace while being timed using a manual stopwatch. Gait speed was calculated by dividing the length of the walkway by the time used from start to finish (meters per seconds).

The CBM scale evaluates high-level balance and mobility on 13 items, with six items performed with both the right and left side of the body, resulting in a total of 19 tasks, scored from zero (“unable to perform”) to five (“performs independently”) and is suggested to represent underlying functional skills required in the community [28]. The tasks are “Unilateral Stance”, “Tandem Walking”, “180 Degree Tandem Pivot”, “Lateral Foot Scooting”, “Hopping Forward”, “Crouch and Walk”, “Lateral Dodging”, “Walking and Looking”, “Running with Controlled Stop”, “Forward to Backward Walking”, “Walk, Look & Carry”, “Descending Stairs”, and “Step-Ups x1 Step” [28]. Higher scores are indicative of better balance and mobility. One item (descending stairs) offers an extra point if participants are able to carry a basket while descending stairs [29]. Individual tasks of the CBM were scored, giving a maximum summary score of 96 points.

### Testing procedure

Data collection took place in movement laboratories at four test sites: (1) Germany (Robert-Bosch Hospital, Stuttgart), (2) Germany (Heidelberg University), (3) Norway (Norwegian University of Science and Technology), and (4) the Netherlands (Vrije Universiteit Amsterdam). All tests were conducted in a single assessment lasting about 1.5–2 h. All participants wore their own low-heeled shoes and were allowed sufficient rest periods at any given time. Trained research staff conducted the assessments.

The CBM testing sessions were videotaped with a digital camera (Sony HDR-CX240E) in full HD, which also recorded the sound, an important feature for the subsequent rating (e.g. to hear the start signal of several tests). Camera height was fixed at 1 m and specific camera positions and angles for each task were predetermined in order to standardize the video recording. The videotaped assessments were scored by two experienced examiners to evaluate interrater reliability. Both raters had on average five years’ experience in assessing balance and mobility using different scales. They received a standardized manual on how to perform the CBM and carried out over 10 assessments. One rater was an exercise scientist (MW), the other a physical therapist (KG). Both raters scored each item independently, being allowed to watch the videos twice, and each of them was blinded to the rating of the other assessor. To determine intrarater reliability, videotaped performance on the CBM was assessed by the same rater a second time three weeks after the first rating.

### Statistical analyses

#### Concurrent validity

Concurrent validity between the CBM and the other balance and mobility tests was assessed using the Spearman’s rank correlation coefficient (*ρ*) since the results of the 8-level balance scale (*p* < .001), errors during 3MTW (*p* < .001), and gait speed test (*p* < .05) were not normally distributed according to the Kolmogorov-Smirnov test. Correlation coefficients of *ρ* < 0.25 were considered as small; 0.25–0.50 as moderate; 0.50–0.75 as good; and > 0.75 as excellent [39].

The determination of the sample size for Spearman’s rank correlation coefficient was based on 2-tailed α ≤ 0.05, statistical power greater than 80%, and a correlation threshold value for the correlation coefficient of 0.50 according to previous validation studies [26, 28, 30]. Based on these assumptions, the minimum sample size required was *n* = 29 [40].

Additionally, exploratory analyses were performed using t-tests in order to examine differences in the CBM performance with regard to the history of falls (fallers vs. non-fallers). T-test was used since the results of the CBM were normally distributed.

#### Inter- and Intrarater reliability and internal consistency

Intraclass Correlation Coefficients (ICC) were utilized for total score interrater (ICC_2,k_) and intrarater (ICC_3,k_) reliability [41]. Desirable standards for reliability coefficients are reported to range from 0.90–0.95 [42]. Inter- and intrarater reliability for each item were evaluated with a generalized kappa statistics [43]. Internal consistency was assessed by Cronbach’s alpha coefficient and item-total correlations, utilizing Spearman’s rank correlation coefficient (*ρ*). Internal consistency with an α > 0.9 was considered as excellent, > 0.8–0.9 as good, > 0.7–0.8 as acceptable, > 0.6–0.7 as questionable, > 0.5–0.6 as poor, and ≤ 0.5 as unacceptable [44].

Item-total correlations, assessed for each individual item and the total CBM score, with a value > 0.2 were considered as satisfactory [45].

#### Ceiling effects

Descriptive statistics included mean, standard deviation, minimum and maximum values of the applied tests. Ceiling effects were analyzed by calculating the percentage of individuals obtaining the highest possible score for the included scales, but only for those assessments which have a clearly predefined minimum or maximum score (CBM, FAB, and 8-level balance scale).

Statistical analysis was performed using IBM SPSS Statistics Version 24.0 (IBM Inc., New York, USA).

## Results

A total of 51 participants aged 66.4 ± 2.7 years (range, 60–70 years; 74.5% female) were tested. Participant characteristics are summarized in Table 1. The number of participants included in the different analyses varied (*N* = 31–51). For the TUG and gait speed test, the first five participants were not assessed. For the participants in Heidelberg (*n* = 16), 3MTW performance was rated only by errors, but not by time. Because time was unavailable, these participants were excluded from statistical analysis on the 3MTW test, resulting in a subsample of 31 participants for which information on time and errors was available.

**Table 1:** Characteristics of the participants (*n* = 51)

	Mean (SD) or % (n)
Country	
Germany (Stuttgart, Heidelberg)	60.8% (31)
Norway (Trondheim)	19.6% (10)
The Netherlands (Amsterdam)	19.6% (10)
Age, years	66.4 (2.7)
Women	74.5 (38)
Body-Mass-Index, kg/m^2^	28.2 (6.0)
Comorbidities, number	1.2 (1.2)
Fallers	19.6% (9)
Number of falls (last 12 months)	0.3 (0.6)

*N* = 51; *SD* Standard Deviation

### Concurrent validity of the CBM

**Fig. 1:**
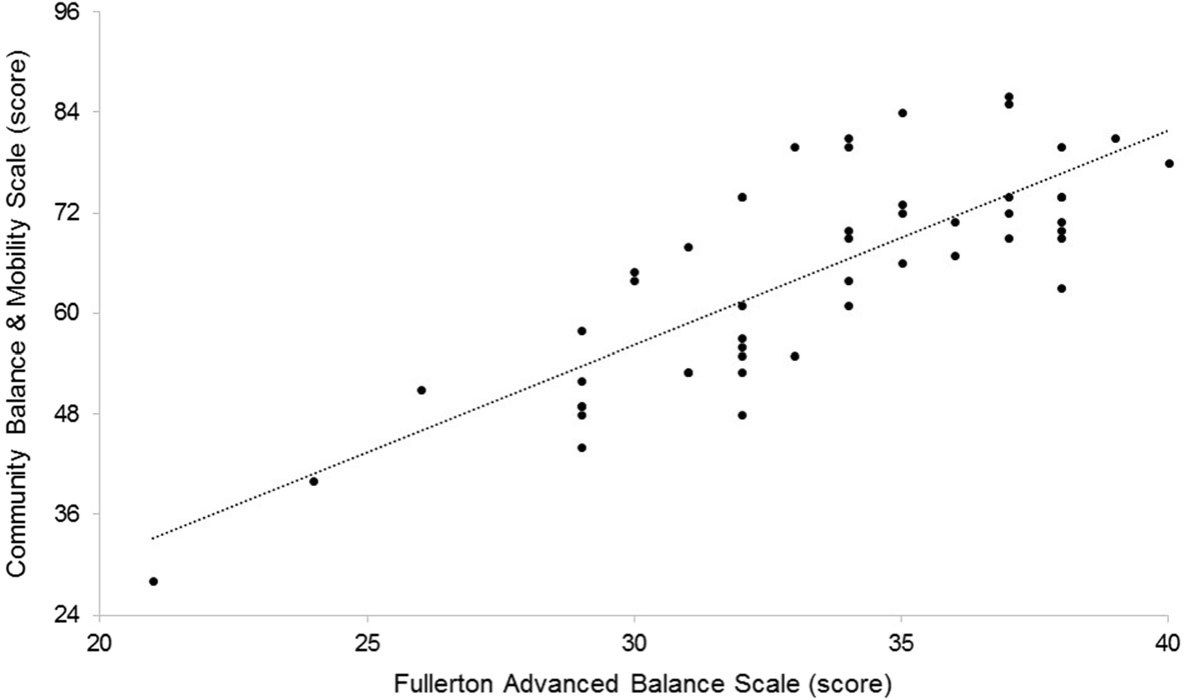
Relationship between CBM total scores and FAB total scores (*n* = 49)

Figure 1 displays the association between CBM and FAB (*ρ* = 0.75; 95% CI = 0.59; 0.85, *p* < .001).

Good correlations were found between CBM and 3MTW errors (*ρ* = − 0.61; 95% CI = − 0.83; − 0.33, *p* < .001). Moderate correlations were found between CBM and gait speed (*ρ* = 0.46; 95% CI = 0.22; 0.66, *p* < .001), TUG (*ρ* = 0.42; 95% CI = − 0.10; − 0.67, *p* = .006), 8-level balance scale (*ρ* = 0.35, 95% CI = 0.04; 0.61, *p* = .013), and 3MTW time (*ρ* = − 0.35; 95% CI = − 0.65; 0.00, *p* = .05) (Table 2). For the discriminative ability of the CBM, no statistically significant differences were identified between fallers (mean score 58.3 ± 14.6) and non-fallers (mean score 66.3 ± 11.8; *p* = .09).

**Table 2:** Correlations between CBM and balance, gait, and walking outcomes

Balance and/or mobility tests	Spearman correlation with CBM score		
	*ρ* (p)	95% CI	*p*-value
FAB scale (score)	0.75	0.59; 0.85	<.001
8-level balance scale (score)	0.35	0.03; 0.61	.013
3MTW test (seconds)^a^	−0.35	−0.65; 0.00	.05
3MTW test (errors) ^a^	−.61	−0.33; − 0.83	<.001
TUG test (seconds)^b^	−0.42	−0.10; − 0.67	.006
Gait speed (cm/seconds)^b^	0.46	0.22; 0.66	<.001

*CBM* Community Balance & Mobility Scale, *FAB* Fullerton Advanced Balance Scale, *3MTW* 3 Meter Tandem Walk, *TUG* Timed Up-and-Go; *ρ* Spearman correlation coefficient, *CI* Confidence Interval

^a^Data reported on 31 participants; ^b^Data reported on 46 participants

### Inter- and intrarater reliability and internal consistency of the CBM

Inter- and intrarater reliability coefficients were excellent with ICC_2,k_ evaluating interrater reliability at 0.97 (95% CI = 0.94–0.98) and ICC_3,k_ evaluating intrarater reliability at 1.00 (95% CI = 0.99–1.00).

Kappa values for individual item reliability are summarized in Table 3. All kappa values were statistically significant (*p* < 0.001). For intrarater reliability, kappa values for 10 of the 19 items were above 0.80 (very good agreement), the other nine were between 0.61 and 0.80 (good agreement). For interrater reliability, two items were above 0.80, ten between 0.61 and 0.80, five between 0.41 and 0.60 (moderate agreement). Two items showed low kappa value of 0.31 and 0.34 respectively [46].

**Table 3:** Inter- and intrarater reliability on item level

	Kappa values (SE)^a^	
Test item (0–5 points)	Intrarater reliability	Interrater reliability
Unilateral stance left	0.94 (0.04)	0.67 (0.08)
Unilateral stance right	0.91 (0.05)	0.78 (0.08)
Tandem walking	0.85 (0.07)	0.74 (0.08)
180° Tandem pivot	0.84 (0.07)	0.55 (0.10)
Lateral foot scooting left	0.91 (0.05)	0.73 (0.08)
Lateral foot scooting right	0.82 (0.07)	0.68 (0.08)
Hopping forward left	0.81 (0.07)	0.59 (0.08)
Hopping forward right	0.78 (0.07)	0.48 (0.09)
Crouch and walk	0.80 (0.08)	0.54 (0.10)
Lateral dodging	0.90 (0.07)	0.67 (0.11)
Walking and looking left	0.75 (0.11)	0.66 (0.12)
Walking and looking right	0.70 (0.12)	0.31 (0.12)
Running with controlled stop	0.75 (0.10)	0.88 (0.08)
Forward to backward walking	0.70 (0.10)	0.34 (0.09)
Walk, look and carry left	0.62 (0.13)	0.49 (0.12)
Walk, look and carry right	0.75 (0.12)	0.68 (0.13)
Descending stairs	0.79 (0.20)	0.85 (0.15)
Step-ups × 1 step left	0.92 (0.05)	0.65 (0.10)
Step-ups × 1 step right	0.91 (0.60)	0.77 (0.10)

*SE* Standard Error

^a^All kappa values are statistically significant with *p*-values = 0.000

Internal consistency was evaluated, with a Cronbach’s alpha of 0.88, indicating good internal consistency.

Item-total correlations ranged from 0.81 (“Hopping forward left”) to 0.28 (“Lateral dodging”). The five items which most strongly correlated with the CBM total score were “Hopping forward left/right”, “Unilateral stance left”, “Forward to backward walking”, and “Lateral foot scooting left” (Table 4).

**Table 4:** Item analyses of the CBM (n = 51)

	Item analyses (*ρ*)
Test item	Item-total correlation^a^ (RO)
Unilateral stance left	0.71 (2)
Unilateral stance right	0.66 (6)
Tandem walking	0.31 (17)
180° Tandem pivot	0.38 (15)
Lateral foot scooting left	0.67 (5)
Lateral foot scooting right	0.53 (11)
Hopping forward left	0.81 (1)
Hopping forward right	0.69 (4)
Crouch and walk	0.36 (16)
Lateral dodging	0.28 (19)
Walking and looking left	0.56 (10)
Walking and looking right	0.51 (12)
Running with controlled stop	0.43 (13)
Forward to backward walking	0.70 (3)
Walk, look and carry left	0.65 (7)
Walk, look and carry right	0.60 (9)
Descending stairs	0.31 (18)
Step-ups × 1 step left	0.61 (8)
Step-ups × 1 step right	0.40 (14)

^a^calculated on the correlation between the item score and the total score; RO, Rank order with 1 = highest value and 17 = lowest value

### Ceiling effects of the CBM and other assessment tools

The participants’ scores are presented in Table 5. The distribution of the CBM scores in the overall sample was negatively skewed, with a median score of 67 points, being higher than the midpoint of the scale (48 points). On the CBM and 8-level balance scale, 0% reached the full score. On the FAB, 2% reached full score.

**Table 5:** Score characteristics of the CBM and other balance and mobility scales

	Mean (SD)	Median	IQR	Minimum	Maximum	Ceiling (100%)	Ceiling (90%)^c^
CBM (0–96 points)	64.7 (12.7)	67.0	55.0–74.0	28.0	86.0	0%	0%
FAB (0–40 points)	33.3 (4.0)	34.0	31.0–37.0	21.0	40.0	2.0%	30.6%
8-level balance (0–7 points)	5.1 (1.1)	5.0	4.0–6.0	2.0	7.0	0%	9.8%
3MTW (time; cont.)^a^	8.4 (2.5)	7.6	6.8–9.4	4.5	16.7	NA	NA
3MTW (errors; cont.) ^a^	.97 (0.32)	0.0	0.0–2.0	0.0	7.0	N/A	N/A
TUG (cont.)^b^	9.1 (1.8)	9.1	7.9–10.6	5.4	13.1	NA	NA
Gait speed (cont.)^b^	128.1 (21.8)	125.0	114.0–142.0	84.3	182.8	NA	NA

*N* = 51; ^a^Data reported on 31 participants; ^b^Data reported on 46 participants;^c^90% of maximum attainable score; SD, Standard Deviation; IQR, Interquartile Range; CBM, Community Balance & Mobility Scale (score); FAB, Fullerton Advanced Balance Scale (score); 3MTW, 3 Meter Tandem Walk; TUG, Timed Up-and-Go test (seconds); Gait speed (cm/seconds); cont., continuous scale

## Discussion

This study is the first to analyze the measurement properties of the CBM in a sample of young-older adults aged 60 to 70 years. As hypothesized, a good correlation with the FAB was found, indicating strong construct validity of the CBM in the target population of young-older adults. Furthermore, moderate to good correlations with other measures suggest that the CBM measures mobility performance (TUG), dynamic steady-state balance control (3 MTW, and gait speed) and static steady-state balance control (8-level balance scale). This is in line with previous studies estimating the measurement properties of the CBM in older adults [26] or those with mild traumatic brain injury [28, 30]. Importantly, the CBM does not show ceiling effects in contrast to other advanced balance scales such as the FAB.

A good correlation was found between the CBM and FAB, showing that both measure a similar construct. Both scales assess performance of more challenging balance tasks, including static, dynamic, proactive, and reactive balance control [28, 30, 32]. The ceiling effect in the FAB may have prevented a higher correlation with the CBM. However, it may also indicate that the tasks within the FAB are not challenging enough to discern difficulties in balance performance in high-functioning older adults [26, 28]. Moreover, the FAB was developed and evaluated to analyze balance impairments in community-dwelling older adults, rather than detecting subtle balance deficits in high-functioning older adults [32]. The correlation with the TUG was moderate (*ρ* = − 0.42), which was lower than expected and lower than reported in a previous study which validated the CBM in older adults [26]. The lower correlation in our sample of young-older adults might be explained by the fact that the TUG is not a highly challenging assessment tool, but rather measures basic functional performance which is typically applied in older adults or patient populations aged ≥70 years [13, 33, 38]. In the present sample, the average time to perform the TUG was 9.1 ± 1.8 s. A study which validated the CBM in older adults reported an average TUG time of 10.4 ± 2.2 s and found a higher correlation between both measures (*ρ* = − 0.69) [26]. The poor discriminative ability of the TUG may have prevented the correlation between the TUG and the CBM from being higher. Recent studies confirm this assumption, showing that the TUG is able to discriminate performances in less healthy, lower-functioning populations (e.g. fallers), but not at discriminating performances in healthy, high-functioning groups [13].

The CBM showed good correlation with 3MTW errors (*ρ* = − 0.61). The 3MTW errors classify a subject based on errors made during a challenging dynamic balance task, which is similar to the classification scheme of the CBM which may explain the good correlation. For 3MTW time, the correlation was lower (*ρ* = − 0.35) as compared to 3MTW errors. This suggests that the quality of task execution (3MTW errors) is more strongly linked to CBM performance as compared to the time of task execution (3MTW time).

Habitual gait speed, a less challenging measure of dynamic balance, showed a moderate correlation with the CBM (*ρ* = 0.46). This suggests that a simple assessment of gait speed, commonly applied in older adults aged ≥70 years [47], may not be sufficient to detect subtle balance deficits in a sample of young-older adults. However, these measurements were intentionally included for comparing the CBM to commonly applied clinical assessment tools and because it has been used in previous validation studies with the CBM in samples of older adults and knee osteoarthritis patients [27, 28].

As expected, a moderate correlation was found between the CBM and the 8-level balance scale (*ρ* = 0.32). The 8-level balance scale is a measure of static steady-state balance control whereas the CBM primarily evaluates dynamic aspects of balance during complex mobility tasks. In line with the present findings, previous studies have reported moderate associations between static and dynamic steady-state balance control, suggesting that both aspects of balance control are partly interrelated, but represent distinct aspects of balance control (e.g. Functional Reach Test vs. gait speed, *r* = 0.08–0.39 [48] or one-leg stand vs. jumping over a hurdle, *r* = 0.05–0.23) [49].

An excellent inter- and intrarater reliability of the CBM total score was found, exceeding the recommended standards of 0.90 to 0.95 for clinical assessments [42]. For the first time, the reliability of the scoring of the single items of the CBM were also evaluated, showing good to very good intrarater reliability [46] for all 19 items. This finding suggests that if the same rater evaluates a participant’s performance on the CBM scale on two separate occasions, high reliability can be expected. For interrater reliability, only five out of the 19 test items had a moderate and two a fair agreement (i.e., “Forward to backward walking” and “Walking and looking right”) [46]. Possible explanations for these two items might be that raters rated individual’s performance differently, such as maintaining straight path versus veering during walking (e.g., “Forward to backward walking”) as well as difficulties to determine for how long the participant’s eyes focused on a point (e.g., “Walking and looking”).

The Cronbach’s alpha as a measure for internal consistency was 0.88. Although it does not exceed the value of 0.90 suggesting redundancies among items [50], further studies should analyze if there are redundant items to design a shortened version of the CBM. As indicated by the results (Table 4), each individual item correlated > 0.20 with the total score, indicating satisfactory internal consistency [45]. On the same note, our findings indicate that future studies with adequate sample sizes should perform a more detailed analysis to purify the CBM. As indicated by Table 4, item-scale correlations for seven items were < 0.50 (“Tandem walking”, “180° Tandem pivot”, “Crouch and walk”, “Lateral dodging”, “Running with controlled stop”, “Descending stairs”, and “Step ups × 1 step right”) which may suggest that their additional value is limited as the cut-off points for internal consistency vary [51–53]. Future studies could determine the underlying factors that represent the CBM construct and eliminate items which cannot be assigned to a factor for purification of the assessment tool. Such factor analyses require a sample size of at least 10 participants per item in the scale [54], which would be 190 participants for the CBM. The development of a shortened CBM has been requested previously [26] and could be of significant benefit as the original version takes 20–30 min to complete.

A limitation of this study is that the sample consists of participants from three countries. While beneficial, cross-national research has limitations. It might be that variation in the performance across the countries could have occurred, despite standardized operating procedures.

Additionally, females were overrepresented in our sample (75%) as compared to the general population aged ≥60 years (56% [55]). However, the sample was too small to perform a stratified analysis for gender. Additionally, the posthoc exploratory analyses for the ability of the CBM to discriminate young-older fallers (mean score 58.3 ± 14.6) from non-fallers (mean score 66.3 ± 11.8) did not reveal statistically significant differences (*p* = .09). A larger sample is needed to evaluate the validity for discriminating fallers from non-fallers.

This cross-sectional study did not allow the determination of responsiveness. Further studies are needed to evaluate the responsiveness of the CBM in the target population.

## Conclusions

This study provides evidence that the CBM is a suitable tool for the assessment of challenging balance and mobility performances in healthy, young-older adults. The CBM tasks represent meaningful everyday performances which are specifically required to ambulate safely within an everyday environment. With trained assessors, the scale is easily administered, requires little equipment, and most importantly, is valid and reliable in the studied target population. Based on the present results, the CBM has been selected as an end point within the EU project PreventIT and is currently used within a randomized controlled trial evaluating a lifestyle-integrated training intervention for preventing functional decline in healthy, young-older adults (registered online; https://clinicaltrials.gov/ct2/show/NCT03065088). The CBM may help to better understand the mechanisms of early balance and mobility decline in young-older adults and inform the development of treatments and intervention programmes aimed at improving early deterioration in balance and mobility, which is in line with the recently updated guidelines for early implementation of neuromotor exercise training in public health approaches [7].

## References

[1] Granacher U, Mühlbauer T, Gruber M (2012). A qualitative review of balance and strength performance in healthy older adults: impact fortesting and training. J Aging Res.

[2] Choy NL, Brauer S, Nitz J (2003). Changes in postural stability in women aged 20 to 80 years. J Gerontol A Biol Sci Med Sci.

[3] Isles RC, Choy NL, Steer M, Nitz JC (2004). Normal values of balance tests in women aged 20–80. J Am Geriatr Soc.

[4] Pollock AS, Durward BR, Rowe PJ, Paul JP (2000). What is balance?. Clin Rehabil.

[5] Garfein AJ, Herzog AR (1995). Robust aging among the young-old, old-old, and oldest-old. J Gerontol B Psychol Sci Soc Sci.

[6] Teno J, Kiel DP, Mor V (1990). Multiple stumbles: a risk factor for falls in community-dwelling elderly; a prospective study. J Am Geriatr Soc.

[7] Bauman A, Merom D, Bull FC, Buchner DM, Fiatarone Singh MA (2016). Updating the evidence for physical activity: summative reviews of the epidemiological evidence, prevalence, and interventions to promote “active aging”. The Gerontologist.

[8] World Health Organization. Global Recommendations on Physical Activity for Health. Geneva: World Health Organization; 2011.

[9] Keadle SK, McKinnon R, Graubard BI, Troiano RP (2016). Prevalence and trends in physical activity among older adults in the United States: a comparison across three national surveys. Prev Med.

[10] Merom D, Pye V, Macniven R, Van der Ploeg H, Milat A, Sherrington C, Lord S, Bauman A (2012). Prevalence and correlates of participation in fall prevention exercise/physical activity by older adults. Prev Med.

[11] Beauchet O, Fantino B, Allali G, Muir SW, Montero-Odasso M, Annweiler C (2011). Timed up and go test and risk of falls in older adults: a systematic review. J Nutr Health Aging.

[12] Berg K, Wood-Dauphinee S, Williams J, Gayton D (1989). Measuring balance in the elderly: preliminary development of an instrument. Physiother Can.

[13] Schoene D, Wu SMS, Mikolaizak S, Menant JC, Smith ST, Delbaere K, Lord SR (2013). Discriminative ability and predictive validity of the timed up and go test in identifying older people who fall: systematic review and meta-analysis. J Am Geriatr Soc.

[14] Tinetti ME (1986). Performance-oriented assessment of mobility problems in elderly patients. J Amer Ger Soc.

[15] Pardasaney PK, Latham NK, Jette AM, Wagenaar RC, Pengsheng N, Slavin MD, Bean JF (2012). Sensitivity to change and responsiveness of four balance measures for community-dwelling older adults. Phys Ther.

[16] Scott V, Votova K, Scanlan A, Close J (2007). Multifactorial and functional mobility assessment tools for fall risk among older adults in community, home-support, long-term and acute care settings. Age Ageing.

[17] Power V, Van De Ven P, Nelson J, Clifford AM (2014). Predicting falls in community-dwelling older adults: a systematic review of task performance-based assessment tools. Physiother Pract Res.

[18] Langley FA, Mackintosh SFH (2007). Functional balance assessment of older community dwelling adults: a systematic review of the literature. Internet J Allied Health Sci Pract.

[19] Guralnik JM, Simonsick EM, Ferrucci L, Glynn RJ, Berkman LF, Blazer DG, Scherr PA, Wallace RB (1994). A short physical performance battery assessing lower extremity function: association with self-reported disability and prediction of mortality and nursing home admission. J Gerontol.

[20] Fleig L, McAllister MM, Chen P, Iverson J, Milne K, McKay HA, Clemson L, Ashe MC (2016). Health behaviour change theory meets falls prevention: feasibility of a habit-based balance and strength exercise intervention for older adults. Psychol Sport Exerc.

[21] Hackney ME, Earhart GM (2010). Effects of dance on gait and balance in Parkinson’s disease: a comparison of partnered and nonpartnered dance movement. Neurorehabil Neural Rep.

[22] Shumway-Cook A, Baldwin M, Polissar NL, Gruber W (1997). Predicting the probability for falls in community-dwelling older adults. Phys Ther.

[23] Hayes KW, Johnson ME. Measures of adult general performance tests: the berg balance scale, dynamic gait index (DGI), gait velocity, physical performance test (PPT), timed chair stand test, timed up and go, and Tinetti performance-oriented mobility assessment (POMA). Arthritis Care Res. 2003;49(S5):S28–S42.

[24] Boulgarides LK, McGinty SM, Willett JA, Barnes CW (2003). Use of clinical and impairment-based tests to predict falls by community-dwelling older adults. Phys Ther.

[25] Rubenstein LZ (2006). Falls in older people: epidemiology, risk factors, and strategies for prevention. Age Ageing.

[26] Balasubramanian CK (2015). The community balance and mobility scale alleviates the ceiling effects observed in the currently used gait and balance assessments for the community-dwelling older adults. J Ger Phys Ther.

[27] Takacs J, Garland SJ, Carpenter MG, Hunt MA (2014). Validity and reliability of the community balance and mobility scale in individuals with knee osteoarthritis. Phys Ther.

[28] Howe J, Inness E, Venturini A, Williams JI, Verrier MC (2006). The community balance and mobility scale - a balance measure for individuals with traumatic brain injury. Clin Rehabil.

[29] Howe JA, Inness E. Community balance & mobility scale. http://www.uhn.ca/TorontoRehab/Health_Professionals/Documents/TR_HCP_SUPP_CBMScale.pdf. Accessed 26 Oct 2016.

[30] Inness E, Howe J, Niechwiej-Szwedo E, Jaglal S, McIlroy WE, Verrier MC (2011). Measuring balance and mobility after traumatic brain injury: validation of the community balance and mobility scale (CB&M). Physiother Can.

[31] Teixeira CS, RC F, Andrade RD, Pereira EF, Dias Lopes LF, Mota CB (2014). Comparison of body balance in active elderly and young adults. Conscientiae Saúde.

[32] Rose DJ, Lucchese N, Wiersma LD (2006). Development of a multidimensional balance scale for use with functionally independent older adults. Arch Phys Med Rehabil.

[33] Podsiadlo D, Richardson S (1991). The timed “up & go”: a test of basic functional mobility for frail elderly persons. J Am Geriatr Soc.

[34] Clemson L, Singh MAF, Bundy A, Cumming RG, Manollaras K, O’Loughlin P, Black D (2012). Integration of balance and strength training into daily life activity to reduce rate of falls in older people (the LiFE study): randomised parallel trial. BMJ.

[35] Ferrucci L, Bandinelli S, Benvenuti E, Di Iorio A, Macchi C, Harris TB, Guralnik JM (2000). Subsystems contributing to the decline in ability to walk: bridging the gap between epidemiology and geriatric practice in the InCHIANTI study. J Am Geriatr Soc.

[36] Nasreddine ZS, Phillips NA, Bédirian V, Charbonneau S, Whitehead V, Collin I, Cummings JL, Chertkow H (2005). The Montreal cognitive assessment, MoCA: a brief screening tool for mild cognitive impairment. J Am Geriatr Soc.

[37] Hernandez D, Rose DJ (2008). Predicting which older adults will or will not fall using the Fullerton advanced balance scale. Arch Phys Med Rehabil.

[38] Mathias S, Nayak US, Isaacs B (1986). Balance in elderly patients: the" get-up and go" test. Arch Phys Med Rehabil.

[39] Portney LG, Watkins MP (2007). Foundations of clinical research: Applications to practice, third edn.

[40] Hulley SB, Cummings SR, Browner WS, Grady D, Newman TB. Designing clinical research: an epidemiologic approach (4th ed. ed.). Philadelphia: Lippincott Williams & Wilkins; 2013.

[41] Shrout PE, Fleiss JL (1979). Intraclass correlations: uses in assessing rater reliability. Psychol Bull.

[42] Nunally JC (1978). Psychometric properties, second edn.

[43] Cohen J (1960). A coefficient for agreement for nominal scales. Educ Psychol Meas.

[44] Cronbach LJ (1951). Coefficient alpha and the internal structure of tests. Psychometrika.

[45] Everitt BS, Skrondal A (2010). The Cambridge dictionary of statistics, 4th edn.

[46] Altman DG (1991). Practical statistics for medical research.

[47] Peel NM, Kuys SS, Kerenaftali K (2013). Gait speed as a measure in geriatric assessment in clinical settings: a systematic review. J Gerontol Ser A Biol Med Sci.

[48] Wernick-Robinson M, Krebs DE, Giorgetti MM (1999). Functional reach: does it really measure dynamic balance?. Arch Phys Med Rehabil.

[49] Sell TC (2012). An examination, correlation, and comparison of static and dynamic measures of postural stability in healthy, physically active adults. Phys Ther Sport.

[50] Streiner DL (2003). Starting at the beginning: an introduction to coefficient alpha and internal consistency. J Pers Assess.

[51] Clark LA, Watson D (1995). Constructing validity: basic issues in objective scale development. Psychol Assess.

[52] Cristobal E, Flavián C, Guinaliu M (2007). Perceived e-service quality (PeSQ). Measurement validation and effects on consumer satisfaction and web site loyalty. Manag Serv Qual.

[53] Loiacono ET, Watson RT, Goodhue DL (2002). WebQual: a measure of website quality. Market Theory Appl.

[54] MacCallum RC, Widaman KF, Zhang S, Hong S (1999). Sample size in factor analysis. Psychol Methods.

[55] He W, Goodkind D, Kowal P, U.S. Census Bureau (2016). An Aging World: 2015. International Population Reports, P95/16–1.

